# Polyethylene Liner Dissociation in the Early Post-Operative Period Following Total Hip Arthroplasty: A Case Report and Literature Review

**DOI:** 10.7759/cureus.25119

**Published:** 2022-05-18

**Authors:** Naga Cheppalli, Akshay Goel, Audry Wassef, Amit W Bhandarkar, Samer Kakish

**Affiliations:** 1 Orthopaedics, Veterans Affairs (VA) Hospital Albuquerque, Albuquerque, USA; 2 Orthopaedic Surgery, Joan C Edwards School of Medicine, Marshall University, Huntington, USA; 3 Department of Orthopaedics, University of New Mexico School of Medicine, Alburquerque, USA; 4 Orthopedics, SSM Health St Mary's Hospital, Centralia, USA; 5 Department of Orthopaedics and Rehabilitation, University of New Mexico, Albuquerque, USA

**Keywords:** total hip arthroplasty: tha, locking mechanism, polyethylene liner, acetabular cup, liner dissociation

## Abstract

Polyethylene liner dissociation (PLD) is a rare but catastrophic complication following total hip arthroplasty (THA). When it occurs in the early postoperative period, it can be easy to miss the diagnosis. Liner dissociation has been reported previously with the Pinnacle^®^ (DePuy), Harris-Galante^®^ (Zimmer), and Trident^®^ (Stryker) acetabular components. To the best of our knowledge, this is the first case reporting PLD in the G7^®^ cup (Zimmer-Biomet). This case report, along with a review of the literature, highlights the clinical presentation, radiological imaging, treatment options, and technical tips to avoid PLD in the early postoperative period.

## Introduction

Modularity in total hip arthroplasty (THA) is very advantageous as it increases the intraoperative flexibility to adjust the length, offset, and anteversion. However, the modularity of the acetabular components can introduce different mechanisms of failure, like backside wear and disassembly of polyethylene from the acetabular shell. Polyethylene liner dissociation (PLD) from the acetabular shell is a rare event, and early diagnosis prevents the need for acetabular component revision. There are several different locking mechanisms designed to fixate polyethylene liners to the acetabular shell. A polyethylene liner is locked into the acetabular shell by locking rings and tabs, taper lock, circular fit, or by increasing the friction fit between the metal and polyethylene component, or by increasing the conformity between the metal shell and PE liner [1]. Multiple factors can contribute to this malfunction of the locking mechanism, and these factors can be classified as technique-related (improper seating and component malposition), design-related, and wear-related or delayed impingement due to changes in hip-spine relationships. R3® acetabular component (Smith and Nephew); Pinnacle® (Depuy), and Harris-Galante® (Zimmer Biomet), have been reported with PLD but to the best of our knowledge, this is the first case reporting PLD in G7® cup (Zimmer Biomet) [2-8]. We also describe the technical tips to avoid this rare complication and the clinical presentation, radiological imaging, and various treatment options described in the literature.

## Case presentation

**Figure 1:**
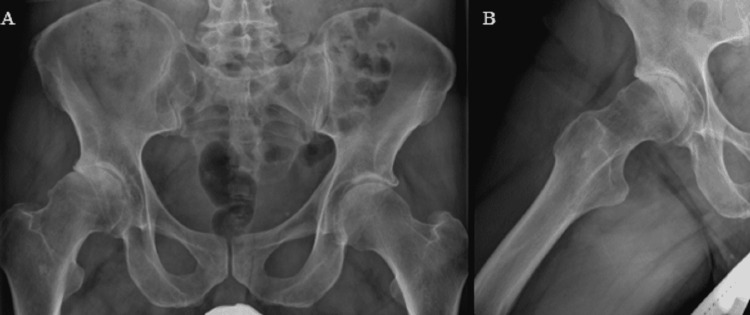
Preoperative images showing right hip osteoarthritis (A) AP pelvis and (B) frog leg lateral view

**Figure 2:**
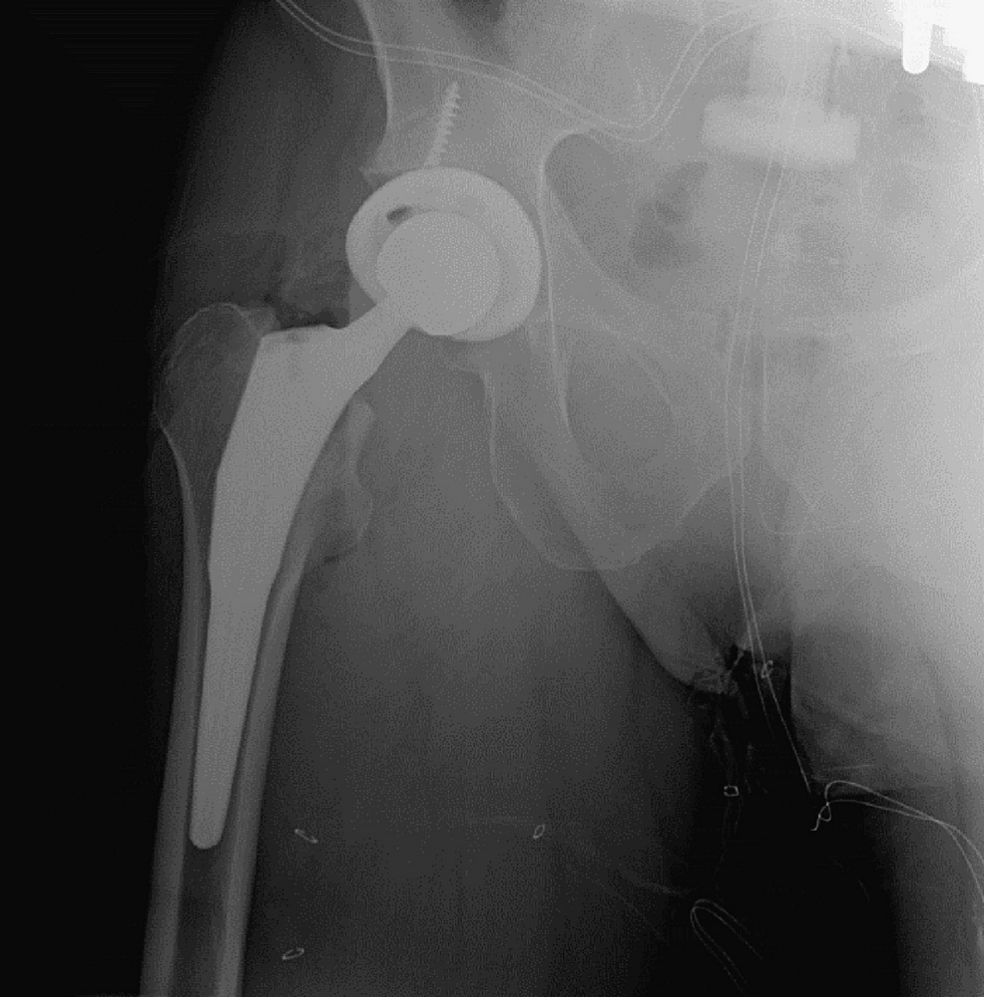
Immediate postoperative image

**Figure 3:**
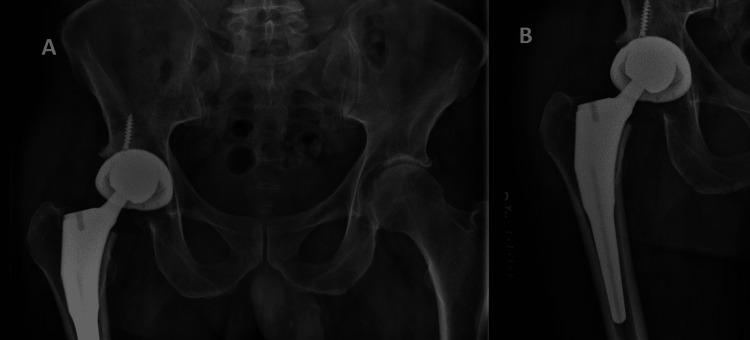
(A) AP radiograph of pelvis and (B) hip showing PLD AP: antero posterior, PLD: polyethylene liner dissociation

**Figure 4:**
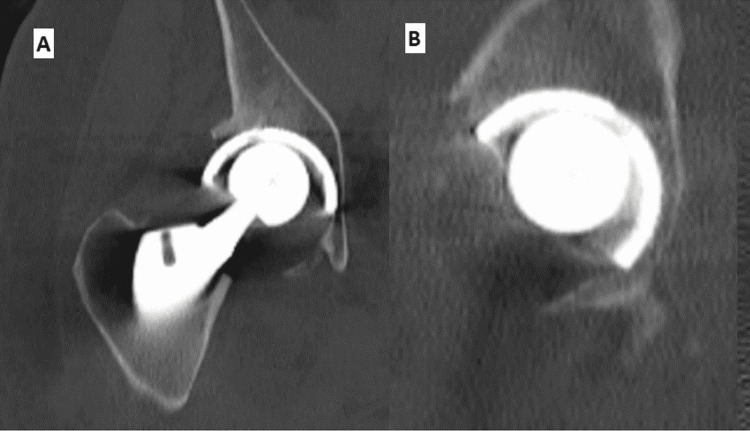
CT scan showing PLD (A) Coronal view and (B) sagittal view

**Figure 5:**
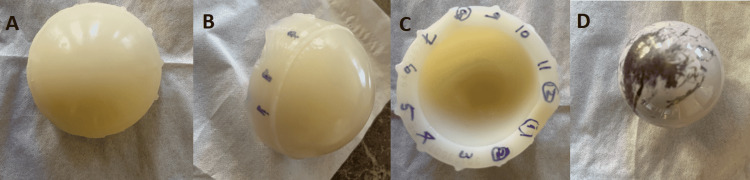
Intraoperative images showing damages to polyethylene and ceramic head (A) showing full profile of damaged poly, (B) arrows indicating the damaged tabs, (C) numbers represented the location of tabs, and (D) damaged ceramic head

A 67-year-old male patient presented to our clinic with significant right hip pain and imaging consistent with end-stage right hip arthritis (Figure 1). The patient elected to proceed with the right THA. A posterior approach was used, and the following implants were placed: Zimmer Biomet G7® OsseoTi acetabular shell size 58 mm, one 6.5 mm x 30 mm length self-tapping screw in the cup. Zimmer Biomet G7 acetabular system high wall 36 mm liner, Taperloc complete® primary femoral stem with a high offset, size 17, and size 36 mm +3 mm modular ceramic head. The patient was discharged after a 48-hour period of observation. Immediate post-operative images revealed a well-articulated femoral head in the acetabular shell (Figure 2). During routine postoperative scheduled follow-up at two weeks for wound inspection, he was doing well except for a grating/squeaking sound. He felt this sound was coming from the right hip. Radiographs demonstrated abnormal eccentric articulation of the femoral head with the acetabular shell, concerning PLD (Figure 3). A CT scan was performed to localize the radiolucent liner (Figure 4). During the revision surgery, the ceramic femoral head was articulated with the metal shell, with the plastic liner along the inferior portion being noted. The ceramic femoral head revealed damage as shown in Figure 5. Some of the tabs of the liner had been damaged, especially those along with the posterior and anterior aspects (tabs located at 8 and 9 o'clock and 12 to 2 PM for the right hip; Figure 5). We ensured the acetabulum shell was not loose and the screws had not backed out. No changes were indicative of damaged grooves in the acetabular shell. The femoral stem was also evaluated for any micro or macro motion with no abnormalities detected. We trialed with a neutral 40 mm liner and a +6 mm 40 mm head and the hip was stable with no impingement. The trial liner was replaced with regular neutral 40 mm polyethylene and a +6 mm, 40 mm ceramic head. Standard closure was obtained with posterior soft tissue repair. Synovial fluid collected during the procedure did not show any evidence of infection.

The patient was discharged two days after surgery and followed up at regular intervals for up to 12 months. He had an uneventful recovery and his Hip Disability and Osteoarthritis Outcome Score (HOOS) improved from 16.3 (at the time of PLD) to 63.6 at 12 months.

## Discussion

PLD is a rare but catastrophic complication with a reported incidence of 15/10,000 [6,9]. There are no reported cases of PLD with G7® (Zimmer Biomet Cup) described previously in the literature. Early diagnosis can prevent complete revision in some scenarios. If PLD occurs, the head can be articulated with an acetabular shell, causing further damage to the liner locking rings and grooves, necessitating complete acetabular revision [10]. If ignored for a longer period, metallosis can set in, causing extensive damage to local tissue. Early in the postoperative period, it is easy to miss the diagnosis as the patient can still bear weight and walk on this articulation, and the pain they experience could be easily confused with post-operative pain. Due to the impact of the COVID-19 pandemic, many postoperative visits are virtual, which increases the chance of missing this complication.

Usually, when PLD occurs, the patient feels a sudden clunk and mild discomfort. The event can be associated with trauma but can also occur spontaneously. In our patient, there was no history of injury or sudden clunk. Patients typically complain of abnormal sounds and squeaking, a grating sensation, relative shortening of the operative leg, or a change in the gait pattern. When radiographs are obtained, they reveal abnormal eccentric articulation with superolateral migration of the femoral head into the shell on the antero-posterior view (AP), and medial migration of the femoral head in the lateral view is usually confirmatory. Unlike PLD associated with polyethylene wear usually, these changes are abrupt. The polyethylene can be seen as a radiolucent ring in some cases (bubble sign/crescent sign). When in doubt, CT scans, MRIs, and ultrasounds are utilized for confirmation of the diagnosis. A CT scan can be helpful to locate the displaced radiolucent polyethylene, however, beam-hardening artifacts can obscure some details. The PE can be seen inferiorly below the capsule or posterior joint recess/greater trochanteric bursa or may be partially associated with the shell. Metal Artifact Reduction Technique Sequence (MARS) can increase the diagnostic confidence relative to routine pulse sequences. MRI with MARS can usually demonstrate polyethylene more clearly than any other investigation. An arthrogram can also help to confirm the diagnosis [11]. Sonographic evaluations have also been described, and they are performed with the patient supine to look for the presence or absence of parallel double hyperechoic lines, which are termed sonographic tram track signs [12]. However, plain radiographs with a clinical history of the sudden onset of a squeaking sound are usually confirmatory in most cases.

If this complication is diagnosed early without damage to the locking mechanism, an isolated liner revision can be performed, making treatment substantially easier. In the case of a late diagnosis, treatment includes either revision of the entire acetabular shell or modification of the causative factors.

Even though we could not conclude the reason for PLD in our case, improper seating of the implant is likely the cause, but other causes, likely femoral neck impingement secondary to the use of high-wall liner, could not be ruled out. PLD could happen during the immediate post-operative period, medium-term (5-10 years), or can be delayed (15 to 20 years) from the primary procedure. The early or immediate PLD is usually technique-related, such as improper seating of the liner, or could be from internal impingement between the neck and the prosthesis liner. The medium-term PLD could be from internal impingement from an abnormal activity or position, or a new onset of impingement from progressive degenerative spine disorder or heterotopic ossification (HO). The late onset of PLD could be a wear-related or a new onset of impingement from DJD of the spine. Backside wear can damage the locking mechanism, resulting in this catastrophic complication.

Technical factors that can impact the incidence of PLD include failing to ensure that the liner is cleared of the soft tissue, blood, or fat at the interface; not being collinear with the shell while impacting poly; damage to the tabs during initial insertion; and prominent screw heads. Occasionally, the acetabular shell can deform during insertion, resulting in improper seating of polyethylene into the shell. Malposition of the acetabular component and femoral component can predispose to impingement leading to PLD. A combination of face-changing or offset acetabular liners with vertically mal-positioned acetabular components can cause impingement, resulting in PLD. The liner should be inspected and palpated circumferentially, and should be manually checked to confirm the locking of the polyethylene into the liner. Some implants have a recess (R 3) along the edge of the acetabular shell to check the proper seating and stability of the locking mechanism. However, it is not present in most of the other existing systems. Most modern acetabular shells have two locking rings and 12 grooves to receive tabs of the liner along the inner surface, and the neutral liner sits flush with the acetabular shell. However, this design feature is not uniformly present in all modern implant companies, and it is important for surgeons to understand the different locking mechanisms (Table 1).

**Table 1:** Review of liner designs from different implants commonly used in North America

Implant company	Number of locking grooves on the shell	Number of tabs on the liner	Relation of liner to shell edge
Pinnacle^®^ (Depuy Synthes, Raynham, MA)	1	6	Flush
G7^®^ (Biomet, Warsaw, IN)	2	12	Flush
R 3^®^ (Smith and Nephew, Wartford, UK)	2	12	Flush
Trilogy^®^ (Zimmer, Erlanger, KY)	2	12	Flush
Trident^®^ (Stryker, Kalamazoo, MI)	2	12	3 mm prominent
Empower^®^ (DJO, Lewisville, TX)	2	12	Flush

In the current case, the linear and horizontal grooves of the articular surface of the shell appeared intact, so we elected to retain the acetabular shell and proceed with head and liner exchange. The original lipped liner and 36 mm ceramic head were swapped with a neutral liner and a 40 mm head. The increased head diameter and neutral liner increased impingement free range of motion. After the new implants were placed, there were no signs of impingement or instability with functional ROM. We also carefully evaluated if there was any micromotion between the liner and the shell before proceeding with this plan. Since this incident happened, 360 degrees of visualization of the liner seating flush with the acetabular shell, and checking the stability of the locking of the liner into the shell with a hemostat at multiple places has become routine during THA.

Overall, this case brings to attention the importance of having a high index of suspicion for PLD. Although it is not a common complication, early detection can significantly reduce the complexity of revision surgery.

## Conclusions

Dissociation of the polyethylene liner from the acetabular shell is a rare complication of THA. When it occurs in the early postoperative period, early recognition is critical in potentially avoiding an acetabular component revision. This study presents a case of PLD in the early postoperative period and discusses the diagnostic challenges in recognizing this complication. Furthermore, we discuss the technical tips to avoid this rare complication and various treatment options described in the literature.
